# Barriers of linkage to HCV viremia testing among people who inject drugs in Georgia

**DOI:** 10.1186/s13011-022-00438-6

**Published:** 2022-03-28

**Authors:** Maia Butsashvili, Tinatin Abzianidze, George Kamkamidze, Lasha Gulbiani, Lia Gvinjilia, Tinatin Kuchuloria, Irina Tskhomelidze, Maka Gogia, Maia Tsereteli, Veronique Miollany, Tamar Kikvidze, Shaun Shadaker, Muazzam Nasrullah, Francisco Averhoff

**Affiliations:** 1 Health Research Union/Clinic NEOLAB, 8 Nutsubidze str., 0177 Tbilisi, Georgia; 2 University of Georgia, 77a M.Kostava str, 0171 Tbilisi, Georgia; 3 TEPHINET, 9 Asatiani street, 0177 Tbilisi, Georgia; 4 Georgian Harm Reduction Network, 24 Shartava str. 3rd floor, apt 6, Tbilisi, Georgia; 5 National Center for Disease Control and Public Health, 99 Kakheti highway, 0198 Tbilisi, Georgia; 6 Medecins Du Monde, 24 Paliashvili str., 0179 Tbilisi, Georgia; 7 Division of Viral Hepatitis, National Center for HIV/AIDS, Viral Hepatitis, STD and TB Prevention, CDC, 1600 Clifton Road, Atlanta, GA 30329-4027 USA

**Keywords:** PWID, Diagnosis, HCV, Elimination, Barriers

## Abstract

**Background:**

People who inject drugs (PWID) in Georgia have a high prevalence of hepatitis C virus antibody (anti-HCV). Access to care among PWID could be prioritized to meet the country’s hepatitis C elimination goals. This study assesses barriers of linkage to HCV viremia testing among PWID in Georgia.

**Methods:**

Study participants were enrolled from 13 harm reduction (HR) centers throughout Georgia. Anti-HCV positive PWID who were tested for viremia (complete diagnosis [CD]), were compared to those not tested for viremia within 90 days of screening anti-HCV positive (not complete diagnosis [NCD]). Convenience samples of CD and NCD individuals recorded at HR centers using beneficiaries’ national ID were drawn from the National HCV Elimination Program database. Participants were interviewed about potential barriers to seeking care.

**Results:**

A total of 500 PWID were enrolled, 245 CD and 255 NCD. CD and NCD were similar with respect to gender, age, employment status, education, knowledge of anti-HCV status, and confidence/trust in the elimination program (*p* > 0.05). More NCD (13.0%) than CD (7.4%) stated they were not sufficiently informed what to do after screening anti-HCV positive (*p* < 0.05). In multivariate analysis, HCV viremia testing was associated with perceived affordability of the elimination program (adjusted prevalence ratio = 8.53; 95% confidence interval: 4.14–17.62).

**Conclusions:**

Post testing counselling and making hepatitis C services affordable could help increase HCV viremia testing among PWID in Georgia.

## Background

Georgia, with a high burden of hepatitis C [1], embarked on a national program in 2015 to eliminate hepatitis C, with an overall goal of 90% reduction in hepatitis C virus (HCV) prevalence by 2020 [2]. At the time of data collection treatment for HCV infection within the framework of the National HCV Elimination Program was available at selected clinics/hospitals throughout the county.

The country has one of the highest rates of injection drug use in the world [3], and 66.2%–92.0% HCV antibody (anti-HCV) prevalence among people who inject drugs (PWID) [4] representing 25% of all HCV cases in the country [5, 6]. Other groups at risk for HCV infection include men who have sex with men (MSM), blood donors and prisoners [2]. Together with injection drug use, blood transfusion, tattoos, frequent dental cleanings, medical injections, dialysis, and having multiple lifetime sexual partners were found as risk factors of HCV transmission in Georgia [7]. Increasing access to hepatitis C screening, care and treatment among PWID is important if Georgia is to be successful in eliminating hepatitis C from the country.

As of April 30, 2019, 56,294 patients had initiated hepatitis C treatment within the program, though there has been a gradual decline in the number of people initiating treatment since late 2016 [8]. Despite extensive national screening activities, including among PWID at harm reduction (HR) centers, nearly half of Georgians with HCV infection are not aware of their HCV status [8]. In addition, among those who have screened positive, a significant proportion have not received viremia testing to confirm active HCV infection, and among those with confirmed viremia, a significant proportion have not initiated antiviral treatment [8].

PWID bear a disproportionate share of the HCV burden in the country [9] and ensuring access to treatment for this marginalized population is critical for achieving elimination in the country. However, barriers to accessing care in the national hepatitis C elimination program among anti-HCV-positive PWID are not well understood. The objective of this study was to assess barriers of linkage to HCV viremia testing among PWID in Georgia.

## Methods

### Settings

All screening, viremia testing and treatment monitoring data are entered into the national elimination program database [8]. Data entry into national elimination database is carried out by clinics/hospitals providing HCV treatment and care services. Access to the database through a unique 11-digit national identification number (NIN) allows for tracking individuals across the whole continuum of care, from screening through treatment and testing for sustained viral response (SVR), i.e. cure.

### Study population

There is a total of 13 HR centers in the country [10] providing Needle and Syringe Program (NSP) services for PWID. Since 2017, anti-HCV test results among PWID who agree to disclose their NIN are recorded in the elimination program database. During 2017–2018, a total of 2780 HR center clients with positive test results for HCV antibody were identified in the program database by their NIN [11].

We compared PWID who had HCV viremia testing diagnosis of hepatitis C (CD) with those who had not (NCD). PWID were defined as people attending Harm Reduction Centers. Complete diagnosis was defined as receipt of HCV viremia testing within 90 days from the date of a positive anti-HCV result recorded in the hepatitis C elimination program database. Convenience samples of CD (*n* = 263) and NCD (*n* = 275) individuals were drawn from 2780 HCV antibody positive individuals who were recorded in national database by HR centers. CD were selected for having a positive anti-HCV result dated between August 1, 2017 and January 31, 2018, while inclusion dates for NCD were between January 1, 2017 and January 31, 2018. The selection period for NCD had to be extended for 7 months to allow for more study participants with available and valid contact information.

### Data collection

We conducted a telephone survey of CD and NCD PWID inquiring about potential barriers to obtaining viremia testing, and/or enrolling in the national hepatitis C elimination program. Interviews were conducted from September 1, 2018 to December 20, 2018. Interviewers were recruited from HR center staff (HIV voluntary counseling and testing counselors) and trained prior to the study. Five attempts were made to reach each study participant. We calculated the proportion of individuals that were not reached via phone and those who refused to participate in the survey. The study protocol was approved by the Institutional Review Board of the Health Research Union (IRB00009520; IORG0005619).

### Survey tool

The telephone questionnaire was developed and pilot tested among five respondents and adapted based on user feedback prior to administration. The questionnaire collected information about demographics and barriers of HCV viremia testing. The social demographic characteristics included age, residence (capital city vs other regions), education, employment, family and personal income. Data collected on HCV included diagnostic test types available in the country, participant’s HCV status and reasons for not being tested. The questionnaire also included questions about current use of HR services (some respondents who were HR center beneficiaries at the time of their anti-HCV test may no longer be receiving HR services at the time of interview), availability of medications, and procedure for enrollment in the program. Participants were also asked about affordability of the program; during the first three years (May 2015 – August 2018) when patients had a co-payment for diagnostic evaluations which was approximately 300 USD at the beginning of the program and later decreased to approximately 100 USD. We also gathered information on respondent preferences for type and source of information about HCV infection.

### Data analysis

Following removal of identifying information, data were entered in Microsoft® Excel at study sites and imported in SPSS v.23 statistical software by the study statistician. Descriptive and bivariate analyses of factors associated with complete diagnosis of hepatitis C were conducted. We calculated unadjusted and adjusted prevalence ratios (PR) with 95% confidence intervals (CI) to assess the associations between CD and NCD using logistic regression models. All variables were included in adjusted analysis.

## Results

### Participation

During 2017–2018, a total of 2780 HR center clients with positive test results for HCV antibody were identified in the program database by their NIN. Out of the 538 PWID drawn by convenience sampling from the elimination program database, 500 individuals agreed to participate in the study (refusal rate of 7.1%): 245 had HCV viremia testing and 255 had not. 59 individuals who didn’t receive HCV RNA testing could not be reached by phone. The participation rates were similar when we compared CD and NCD.

### Demographic characteristics of study participants

Overall, 91.2% of study subjects were males, 81.4% were older than 35 years, 63.8% were unemployed, and 34.9% had a university/college degree. No significant differences were found between the two groups (CD and NCD) by age, gender, residence, family income, employment status, education level, and confidence/trust in the hepatitis C elimination program (Table 1).

**Table 1:** Comparison of complete diagnosis (CD) and not complete diagnosis (NCD) among people who inject drugs by different characteristics, Georgia

Characteristic	Complete diagnosis (CD)		Not complete diagnosis (NCD)			Prevalence Ratio (PR) and 95% CI	Adjusted Prevalence Ratio (aPR) and 95% CI
	**N**	**%**	**N**	**%**			
**Gender**							
Male	224	91.4	232	91.0		1.01 (0.95–1.06)	1.09 (0.380–3.18)
Female	21	8.6	23	9.0		1	1
**Age**							
≤ 35	38	15.5	55	21.6		1.20 (0.99–1.46)	1.23 (0.56–2.69)
> 35	207	84.5	200	78.4		1	1
**Residence (district)**							
Tbilisi	105	42.9	127	49.8		1	1
Regions	140	57.1	128	50.2		1.14 (0.97–1.34)	0.8 (0.36–1.80)
**Level of education**^**a**^							
University/Post-graduate	80	33.3	91	36.4		1	1
Other	160	66.7	159	63.6		1.05 (0.92–1.19)	1.96 (0.94–4.11)
**Employment**^**a**^							
Employed	92	38.0	87	34.4		1	1
Unemployed	150	62.0	166	65.6		0.95 (0.83–1.08)	1.26 (0.65–2.57)
**Family income (per month)**^**a**^							
≤ 1000 GEL	122	87.8	133	90.5		0.97 (0.89–1.05)	1.21 (0.42–3.52)
> 1000 GEL	17	12.2	14	9.5		1	1
**Affordability of HCV Elimination Program**^**a**^							
Yes	182	75.5	105	41.8		1.81 (1.53–2.12)	8.53 (4.14–17.62)
No	59	24.5	146	58.2		1	1
**Who notified you about the screening test result?**^**a**^							
Person who conducted the test	64	33.2	56		28.3	1.17 (0.87–1.58)	1.02 (0.67–1.23)
Physician/Consultant	129	66.8	142		71.7	1	1
**Do you feel that you received sufficient information to know what to do next after screening test?**^**a**^							
Yes	226	92.6	220		87.0	1.07 (1.01–1.13)	0.61 (0.17–1.99)
No	18	7.4	33		13.0	1	1
**Trusting HCV elimination program**^**a**^							
Yes	231	95.1	236		92.5	1	1
No	12	4.9	19		7.5	0.66 (0.33–1.33)	0.73 (0.13–4.09)
**Do you currently use the services in HR centers?**^**a**^							
Yes	111	48.7	90		37.3	1.31 (1.07–1.61)	1.21 (0.91–1.52)
No	117	51.3	151		62.7	1	1
**Would it be more comfortable if HCV treatment were available in HR centers?**^**a**^							
Yes	169	84.9	190		86.4	0.98 (0.91–1.06)	0.65 (0.32–1.29)
No	30	15.1	30		13.6	1	1

^a^Missing values not shown

Abbreviations: *CI* confidence interval, *HCV* hepatitis C virus, *GEL* Georgian lari, *HR* harm reduction

### Knowledge of HCV and elimination program

Most of the study participants (98.2%) were aware of their HCV exposure. Of the surveyed individuals, 391 (78.2%) reported that they had been informed about HCV test results from a health care worker (HCW).

When we looked at reasons why the NCD group did not receive viremia testing, 21.7% stated that the reason was cost of the testing, 42.9% of the NCD group stated that a free laboratory test would improve enrollment in the program and more than 20% of respondents indicated they were not informed about their test result by HCW. A quarter (24.5%) of NCD participants expressed willingness to receive more information about HCV infection and the Hepatitis C Elimination Program, preferring television and the internet as sources of information.

### Association of complete diagnosis of hepatitis C with different factors

In bivariate analysis, 13.0% of NCD compared to 7.4% of CD stated they did not receive sufficient information to know what to do next after their positive screening test (PR = 1.07; 95% CI: 1.01–1.13). More NCD (58.2%) than CD (24.5%) reported that enrollment was not affordable (PR = 1.81; 95% CI: 1.53–2.12). Ongoing engagement in HR services was associated with complete diagnosis (PR = 1.31; 95%CI: 1.07–1.61). Nearly 85% of CD and 86.4% of NCD think that it would be more comfortable if HCV treatment were available at HR centers (PR = 0.98; 95% CI: 0.91–1.06). However, in multivariate analysis, the only independent predictor of complete diagnosis was affordability of the program (adjusted PR = 8.53; 95% CI: 4.14–17.62) (Table 1).

## Discussion

This is the first quantitative study in Georgia to examine barriers to HCV treatment among PWID, which represents a priority for the program. Mathematical modeling suggests that for countries with a large burden of injection drug use, HCV treatment for PWID is critical to achieving HCV elimination [12]. We found that barriers of linkge to HCV viremia testing anti-HCV positive PWID include perceived high cost of care and a lack of information on what to do after a positive screening.

The eligibility criteria for HCV-infected individuals to enroll in the hepatitis C elimination program in Georgia are simple i.e., a person must be a citizen of Georgia aged ≥ 18 years. At the beginning of the program (April 28, 2015 to June 9, 2016) only patients with advanced liver fibrosis level were eligible for treatment [13]. However, since June 2016, the program has been expanded to all HCV-viremic individuals regardless of disease severity [14]. This expansion resulted in an increase in the number of enrolled individuals, but enrollment gradually declined after its peak in September 2016. Exact reasons for this decline are not known but high cost of diagnostics earlier in the program may be attributed to this decline. In our analysis, affordability of the program was the only independent predictor of complete diagnosis.

Although all PWID interviewed were utilizing HR services at the time they were screened, more than half of study participants were no longer receiving HR services at the time of interview. Disengagement with HR services was one of the factors associated with the low rate of HCV viremia testing. Research suggests engagement in opioid substitution therapy and other HR services increases the rate of HCV viremia testing diagnosis among PWID [15]. Our data seem to correlate with this finding.

One important finding of this study was that more than 20% of respondents did not indicate they were informed of their test results by their HCW. Ensuring counseling to communicate screening test results and the need and procedure for follow-up viremia testing among those with positive screening results may result in increased rate of HCV viremia testing. According to a recent study conducted among PWID receiving methadone substitution treatment (MST) in Georgia, more than 75% of MST patients who had HCV viremia testing initiated and 95% completed antiviral treatment within the National HCV Elimination Program [16]. Further, standardized counseling procedures need to be developed and implemented at all HR facilities to inform anti-HCV-positive patients about the need for HCV viremia testing to improve the rate of complete diagnosis.

Several studies indicate different barriers for HCV infection care continuum among PWID, including refusal to engage with healthcare workers due to stigma associated with drug use, [17–19] mistrust of the healthcare system [20], low level of perception about the need for treatment, and concerns about waiting periods and drug withdrawal [21]. Unlike these studies, in our study the main barriers of linkage to HCV viremia testing among PWID were a lack of information about further steps after receiving a positive HCV antibody test and unawareness about the availability of free diagnosis and treatment. Interventions to improve HCV care contimuun among PWID have been identified by different studies. One study found increased enrollment rates and adherence to treatment among PWID with advanced liver fibrosis level when hepatitis C screening is done on-site at the HR center and when peer navigation services are available [6]. A systematic review revealed that linkage to care among PWID was facilitated by referral for hepatitis C assessment and scheduling of appointment with specialist physician [22]. In addition, peer support models were successfully used to increase uptake of hepatitis C treatment services [23].

Other services, such as on-site viremia testing and treatment have also been shown to be effective [24], and could help the country to reach elimination.

This study is subject to limitations. Firstly, our findings are not representative of all PWID in the country. We were unable to include those PWID in our study who were not enrolled in HR services at the time of anti-HCV testing and who did not agree to provide their NIN to be registered in the elimination program database. Individuals not enrolled in HR services are likely among the hardest to reach, and we were not able to survey this population. Secondly, these findings are self-reported and are subject to recall and social desirability biases. Another limitation of the study is the different selection periods for NCD and CD groups (longer selection period for NCD), which could lead to information bias, as awareness of the study participants could change over time.

## Conclusion

Post testing counselling and making hepatitis C services affordable could help increase the rate of HCV viremia testing among PWID in Georgia. Reducing barriers for PWID is critical, and data from this study may be useful for other countries with a high HCV prevalence and where injection drug use is a major route of HCV transmission.

## Data Availability

All data generated or analysed during this study are included in this published article.

## Abbreviations

HCV: Hepatitis C virus
HIV: Human Immunodeficiency Virus
HR: Harm Reduction
CD: Complete diagnosis
NIN: National Identification Number
NCD: Not complete diagnosis
NSP: Needle and Syringe Program
PWID: People Who Inject Drugs
TAG: Technical Advisory Group
USD: United States Dollar
